# Mathematical modeling and stability analysis of Pine Wilt Disease with optimal control

**DOI:** 10.1038/s41598-017-03179-w

**Published:** 2017-06-08

**Authors:** M. A. Khan, K. Ali, E. Bonyah, K. O. Okosun, S. Islam, A. Khan

**Affiliations:** 1 Department of Mathematics, Abdul Wali Khan University, Mardan, Khyber Pakhtunkhwa, 23200 Pakistan; 2 Department of Mathematics, Abdul Wali Khan, University Mardan, Khyber Pakhtunkhwa, Pakistan; 3 Department of Mathematics and Statistics, Kumasi Technical University, Kumasi, Ghana; 4 Department of Mathematics, Vaal University of Technology, X021, Vanderbijlpark, 1900 South Africa; 5 Department of Physics, Abdul Wali Khan University Mardan, 23200 Mardan, Pakistan

## Abstract

This paper presents and examine a mathematical system of equations which describes the dynamics of pine wilt disease (PWD). Firstly, we examine the model with constant controls. Here, we investigate the disease equilibria and calculate the basic reproduction number of the disease. Secondly, we incorporate time dependent controls into the model and then analyze the conditions that are necessary for the disease to be controlled optimally. Finally, the numerical results for the model are presented.

## Introduction

The forest has a significant role in human life, therefore, it is necessary to put in place safety measure in order to protect the trees from being infected with diseases. The trees do not only provide greenery to the environment but also provide pleasant atmosphere for human community. The pine wilt disease (PWD) is one of the major threat to the forest and ecosystem. It is one of the dramatic disease, that kills, pine trees within a very short time and one of the symptom of this diseases is the red dish brown foliage. The North American native pinewood nematode (Bursaphelenchus xylophilus) which is vectored by *Monochamus alternatus* (Japanese pine sawyer beetle, a species that transmits the nematode to healthy trees) is one of the causes of pine wilt disease^1–5^. This disease has been known since 1900s in Japan^6^, and 1980s^7^ in China and causes great economic and environmental loss to ecosystems worldwide.

This disease has three organisms: a pinewood nematode, a gymnosperm host, and an insect vector. During primary transmission, dauer juveniles (JIV stage) of *Bursaphelenchus xylophilus* are carried phoretically in the tracheae of their beetle host to young twigs of susceptible trees, where they enter through resin canals in wounds made during maturation feeding by the insect^6, 8^. The dead or dying conifer is a suitable breeding host for the next generation of Monochamus spp. vectors. Nematodes brought into the conifer during oviposition of the beetle (secondary transmission) will moult from the dauer juvenile stage and enter the propagative phase to grow and reproduce on secondary fungi that are present as the pine host dies. Then the cerambycid eggs hatch and develop through several larval instars while producing galleries, at first in the inner bark, cambium and outer sapwood and later in deeper woody tissue^8–10^. The pine wilt disease is caused by *Bursaphelenchus xylophilus* ^11, 12^ and the vector is the pine sawyer beetles (*Monochamus alternatus*). The nematode are scattered by the vector beetles over pine healthy trees from space, while in the vector population the direct transmission can happen while vectors are mating^13, 14^. During breeding, mature beetles use healthy tree twigs mainly for feeding purposes while they focus on the infected trees only for copulation and oviposition^15^. The transmission of *Bursaphelenchus xylophilus* horizontally between heterosexual vectors promotes multiple infections. The PWD’s first outbreak in 1905 occurred in Japan, the disease has spread throughout the country in the 1970s and only excluding the northern parts of Japan^16^. After a decade, PWD had spread to many parts of Asia, such as China, Taiwan, Hong Kong and South Korea and in 1999, the disease hit Europe (Portugal)^17^. Today, PWD has become one of the major threats all over the world to forests ecosystems.

Pine wilt disease is very disastrous and can within weeks kill affected trees. The death of the trees are actually caused by a microscopic pine wood nematodes (*Bursaphelenchus xylophilus*) and the management of pine wilt disease is limited to prevention primarily. There are no cures for the disease once a susceptible tree becomes infected with pinewood nematode and these parasitic nematodes do not associate with the plant roots, but to the aboveground parts of the pine trees. The spread of the nematode from trees to trees occur via the pine sawyer beetle (Monochamus spp.) as they feed on healthy trees branches, the nematodes would then emerge and thereafter enter through the feeding wounds caused by the beetles into the trees. Infected trees thereafter are killed by nematodes feeding on cells surrounding the resin ducts.

During oviposition (egg laying), the adult sawyers are by preference only attracted to the dead or dying trees. And the tree age also influences the risk of pine wilt. Trees that are 10 years and above are the most affected, beetles feed and infest one or more trees with nematodes and then move to other susceptible area. But very often the disease only affects a tree in a group. This is due to the nematodes movement, which is not based on physical contact or water or through grafts of roots^18, 19^.

Some mathematical models have been presented on the dynamics of PWD. A mathematical model presented in ref. 20, examined the stability of pine wild disease and application of optimal control technique, where the population of pine trees were divided into two categories, that is, susceptible pine trees and infected pine trees, while the vector population (beetles) were divided into two classes; susceptible vector and infected vector^20^. Also, K. S. Lee and A. A. Lashari^21^ introduced a mathematical model that incorporated the exposed class in the pine trees population with a detailed discussion made on the stability and optimal control of PWD. Also, M. Ozair presented a mathematical model on the dynamics of PWD by dividing the host pine trees and vector beetles into susceptible and infected classes with nonlinear incidence and horizontal transmission^22^. Recently, K. S. Lee and D. Kim introduced a mathematical model that describe the dynamics of PWD by presenting its global stability with nonlinear incidence rates^23^.

It is necessary to make the community and forest safe from infectious diseases, with different control measure used. Such as optimal control technique and mathematical models are widely used to understand the complicated nonlinear phenomena of infectious diseases with different purposes^24–27^ which is helpful in analyzing biological models. In the literature, various articles on the dynamics of infectious disease with optimal control strategies are presented^28–31^ and the reference there in.

The aim of this paper is to present a mathematical model on the dynamics of Pine trees and vector (beetles) population. We first presented the detail mathematical study of the model, which is the local and global stability, asymptotical stability and backward bifurcation phenomena. Then, we applied the optimal control technique to minimize the population of exposed pine trees, infected pine trees, susceptible vector, exposed vector and infected vector (beetles) as well as to maximize the population of susceptible pine trees. Different control strategies have been presented in order to reduce infection in the population of pine trees.

In the next, section Basic Model Formulation we give a detail analysis about the mathematical formulation of PWD. In section Stability analysis disease free a brief mathematical results are presented for disease free case and a backward bifurcation analysis. The stability analysis of the model at endemic equilibrium is presented in Section stability endemic equilibrium. Further, we apply the optimal control technique in section Optimal control problem while the numerical results and conclusion are presented in sections Numerical results and Conclusion respectively

## Basic Model Formulation

The total population of pine wood trees is denoted by *N*(*t*) and we subdivide into four subclasses; the pine trees that are susceptible, *S*_*H*_(*t*), pine trees that are already exposed, *E*_*H*_, and the pine trees that are infected *I*_*H*_ at any time *t*, with ${N}_{H}(t)={S}_{H}(t)+{E}_{H}(t)+{I}_{H}(t)$.

The total population of vector (beetles) is denoted by *N*_*V*_, which is categorized further into subclasses, namely, the susceptible beetles, *S*_*V*_, the exposed vector beetles, *E*_*V*_(*t*) (which not carrying pinewood nematode), and infected vector beetles, *I*_*V*_(*t*)(which have the ability to carry pinewood nematode) at any time *t*, with ${N}_{V}(t)={S}_{V}(t)+{E}_{V}(t)+{I}_{V}(t)$.

We are not interested to include the class *R*_*H*_(*t*) for pine wood trees population, this is because the infected pine wood tree dies in a year or may in a subsequent next years.

**Figure 1:**
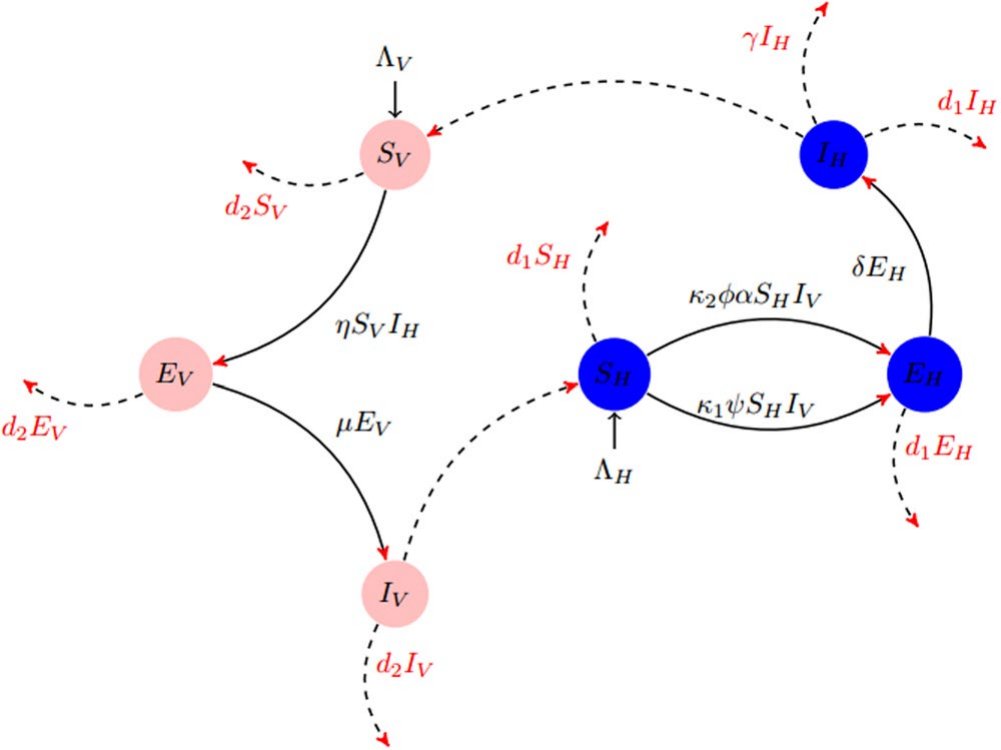
Flow chart for the transmission for the Pine wilt disease PWD.

With the above assumptions, we present the model and their schematic diagram in Fig. 1 is follows:

1 $$\{\begin{array}{rcl}\frac{d{S}_{H}}{dt} & = & {{\rm{\Lambda }}}_{H}-{\kappa }_{1}\psi {S}_{H}{I}_{V}-{\kappa }_{2}\varphi \alpha {S}_{H}{I}_{V}-{d}_{1}{S}_{H},\\ \frac{d{E}_{H}}{dt} & = & {\kappa }_{1}\psi {S}_{H}{I}_{V}+{\kappa }_{2}\varphi \alpha {S}_{H}{I}_{V}-({d}_{1}+\delta ){E}_{H},\\ \frac{d{I}_{H}}{dt} & = & \delta {E}_{H}-({d}_{1}+\gamma ){I}_{H},\\ \frac{d{S}_{V}}{dt} & = & {{\rm{\Lambda }}}_{V}-\eta {S}_{V}{I}_{H}-{d}_{2}{S}_{V},\\ \frac{d{E}_{V}}{dt} & = & \eta {S}_{V}{I}_{H}-({d}_{2}+\mu ){E}_{V},\\ \frac{d{I}_{V}}{dt} & = & \mu {E}_{V}-{d}_{2}{I}_{V}\mathrm{.}\end{array}$$

The parameter Λ_*H*_ denotes the recruitment rate of pine trees that are susceptible and the parameter *κ*_1_ stands for the contact rate during maturation. The average number of contacts during maturation per day with vector adult beetles is denoted by *ψ*. The mass action term *κ*_1_ *ψS*_*H*_ *I*_*V*_ represents the incidence rate. The parameter *κ*_2_, denote the probability that a nematode is being transmitted through oviposition by an infected beetle, and the average number of contacts per day when adult beetles oviposit is denoted by *ϕ*. The probability that pine trees that are susceptible cease oleoresin exudation without infected by the nematode is represented by *α*. We show the transmission through oviposition by *κ*_2_ *ϕα* and hence, *κ*_2_ *ϕαS*_*H*_ *I*_*V*_ denotes the number of new infections. The rate of progression from pine trees that are exposed to trees that are infected and the natural death rate of pine trees respectively denoted by *δ* and *d*_1_.

The vector pine beetle emergence rate is denoted by Λ_*V*_, while *η* measure the rate at which adult beetles that are escaping from dead trees carry the PWN with them and so the transmission via this route is denoted by *ηS*_*V*_ *I*_*H*_. The transfer rate from *E*_*v*_ to *I*_*v*_, the natural death rate and disease induced death rate for the vector beetles population are respectively denoted by *μ*, *d*_2_ and *γ*. The model (1) presents the pine wood trees and vector beetles populations, and it is understood that all the variables involved with parameters are nonnegative for nonnegative initial conditions.

So, the initials conditions for the model (1) is follows as: ${S}_{H}\mathrm{(0)}={S}_{H}^{o}$, ${E}_{H}\mathrm{(0)}={E}_{H}^{o}$, ${I}_{H}\mathrm{(0)}={I}_{H}^{o}$, ${S}_{V}\mathrm{(0)}={S}_{V}^{o}$, ${E}_{V}\mathrm{(0)}={E}_{V}^{o}$ and ${I}_{V}\mathrm{(0)}={I}_{V}^{o}$.

The total population of pinewood trees is $d{N}_{H}/dt={{\rm{\Lambda }}}_{H}-{d}_{1}{N}_{H}$. The total population of vector beetles is $d{N}_{V}/dt={{\rm{\Lambda }}}_{V}-{d}_{2}{N}_{H}$.

When *t* → ∞, the total dynamics of pine wood trees and beetles approaches $({N}_{H}(t),{N}_{V}(t))\to ({{\rm{\Lambda }}}_{H}/{d}_{1},{{\rm{\Lambda }}}_{V}/{d}_{2})$. Thus, the biological feasible region for model (1) is

$$\begin{array}{rcl}{\rm{\Psi }} & = & \{({S}_{H},{E}_{H},{I}_{H},{S}_{V},{E}_{V},{I}_{V})\in {R}_{+}^{6}|0\le {S}_{H}+{E}_{H}+{I}_{H}\le \frac{{{\rm{\Lambda }}}_{H}}{{d}_{1}},0\le {S}_{V}\\  &  & +\,{E}_{V}+{I}_{V}\le \frac{{{\rm{\Lambda }}}_{V}}{{d}_{2}}\}\end{array}$$

which is positively invariant and the global is attracted in ${\rm{\Psi }}$.

The disease free equilibrium of the system (1) denoted by ${{\mathscr{K}}}_{0}$ and is given by ${{\mathscr{K}}}_{0}=({S}_{H}^{0},0,0,{S}_{V}^{0},0,0)$.

### Basic reproduction number

Here, we will compute the basic reproduction number ${ {\mathcal R} }_{0}$ of system (1). The concept of next generation matrix and basic reproduction number in refs 32, 33 will be used to obtain ${ {\mathcal R} }_{0}$ for the proposed model (1). Hence, we define the new vector $\bar{x}=({E}_{H},{I}_{H},{E}_{V},{I}_{V})$ contains only the infected variables. Consider the following system:

2 $$\{\begin{array}{rcl}\frac{d{E}_{H}}{dt} & = & {\kappa }_{1}\psi {S}_{H}{I}_{V}+{\kappa }_{2}\varphi \alpha {S}_{H}{I}_{V}-({d}_{1}+\delta ){E}_{H},\\ \frac{d{I}_{H}}{dt} & = & \delta {E}_{H}-({d}_{1}+\gamma ){I}_{H},\\ \frac{d{E}_{V}}{dt} & = & \eta {S}_{V}{I}_{H}-({d}_{2}+\mu ){E}_{V},\\ \frac{d{I}_{V}}{dt} & = & \mu {E}_{V}-{d}_{2}{I}_{V}\mathrm{.}\end{array}$$

In the computation of ${ {\mathcal R} }_{0}$ ^32^, the necessary matrices involved are obtained as follows:

$$F=(\begin{array}{cccc}0 & 0 & 0 & {S}_{H}^{0}(\psi {\kappa }_{1}+\alpha \varphi {\kappa }_{2})\\ 0 & 0 & 0 & 0\\ 0 & \eta {S}_{V}^{0} & 0 & 0\\ 0 & 0 & 0 & 0\end{array}),\,V=(\begin{array}{cccc}\delta +{d}_{1} & 0 & 0 & 0\\ -\delta  & \gamma +{d}_{1} & 0 & 0\\ 0 & 0 & \mu +{d}_{2} & 0\\ 0 & 0 & -\mu  & {d}_{2}\end{array}).$$

The inverse of *V* equals

$${V}^{-1}=(\begin{array}{cccc}\frac{1}{\delta +{d}_{1}} & 0 & 0 & 0\\ \frac{\delta }{(\gamma +{d}_{1})(\delta +{d}_{1})} & \frac{1}{\gamma +{d}_{1}} & 0 & 0\\ 0 & 0 & \frac{1}{\mu +{d}_{2}} & 0\\ 0 & 0 & \frac{\mu }{{d}_{2}(\mu +{d}_{2})} & \frac{1}{{d}_{2}}\end{array}).$$

Thus, the next generation matrix of system (2) is

$$F{V}^{-1}=(\begin{array}{cccc}0 & 0 & \tfrac{\mu {S}_{H}^{0}(\psi {\kappa }_{1}+\alpha \varphi {\kappa }_{2})}{{d}_{2}(\mu +{d}_{2})} & \tfrac{{S}_{H}^{0}(\psi {\kappa }_{1}+\alpha \varphi {\kappa }_{2})}{{d}_{2}}\\ 0 & 0 & 0 & 0\\ \tfrac{\delta \eta {S}_{V}^{0}}{(\gamma +{d}_{1})(\delta +{d}_{1})} & \tfrac{\eta {S}_{V}^{0}}{(\gamma +{d}_{1})} & 0 & 0\\ 0 & 0 & 0 & 0\end{array}).$$

So the basic reproduction number is

$${ {\mathcal R} }_{0}=\rho (F{V}^{-1})=\sqrt{\frac{\delta \eta \mu {{\rm{\Lambda }}}_{H}{{\rm{\Lambda }}}_{V}({\kappa }_{1}\psi +{\kappa }_{2}\varphi \alpha )}{{d}_{1}{d}_{2}^{2}(\gamma +{d}_{1})(\delta +{d}_{1})({d}_{2}+\mu )}},$$

where *ρ*, ${S}_{H}^{0}=\frac{{{\rm{\Lambda }}}_{H}}{{d}_{1}}$, ${S}_{V}^{0}=\frac{{{\rm{\Lambda }}}_{V}}{{d}_{2}}$ represent respectively, the spectral radius, disease free equilibrium of trees and disease free equilibrium of vector. In the following, we show that ${ {\mathcal R} }_{0}$ is the key threshold parameters whose values completely characterize the global dynamics of system (1).

## Stability Analysis of Disease free Equilibrium

Let

$$M=F-V=(\begin{array}{cccc}-(\delta +{d}_{1}) & 0 & 0 & {S}_{H}^{0}(\psi {\kappa }_{1}+\alpha \varphi {\kappa }_{2})\\ \delta  & -(\gamma +{d}_{1}) & 0 & 0\\ 0 & \eta {S}_{V}^{0} & -(\mu +{d}_{2}) & 0\\ 0 & 0 & \mu  & -{d}_{2}\end{array}).$$

Define $s(M)=max\{Re\lambda :\lambda \,is\,an\,eigenvalue\,of\,M\}$, so *s*(*M*) is a simple eigenvalue of *M* with a positive eigenvector^34^. It follows from ref. 32, that two equivalences hold: ${ {\mathcal R} }_{0} > 1\iff s(M) > 0$, ${ {\mathcal R} }_{0} < 1\iff s(M) < 0$.

**Theorem 0.1**. *If* ${ {\mathcal R} }_{{0}} < {1}$, *then the disease-free equilibrium* ${{\mathscr{K}}}_{{0}}$ *is locally asymptotically stable on* ${{\Psi }}_{{1}}$.

**Proof:** To show this results, we check the hypothesis present in ref. 32, namely (*A*_1_)–(*A*_5_). The hypothesis (*A*_1_)–(*A*_4_) can be verified easily, while *A*_5_ could be satisfied if all the eigenvalues of the 6 × 6 matrix

$${J|}_{{K}_{0}}=(\begin{array}{cc}M & 0\\ {J}_{3} & {J}_{4}\end{array}),$$

have negative real parts, where *J*_3_ = −*F*,

$${J}_{4}=(\begin{array}{cccc}-(\delta +{d}_{1}) & 0 & 0 & 0\\ \delta  & -(\gamma +{d}_{1}) & 0 & 0\\ 0 & 0 & -(\mu +{d}_{2}) & 0\\ 0 & 0 & \mu  & -{d}_{2}\end{array}).$$

Calculated the eigenvalues of *J*_4_,

$$s({J}_{4})=max(\{-\gamma -{d}_{1},-(\delta +{d}_{1}),-({d}_{2}+\mu ),-{d}_{2}\}) < 0.$$

If ${ {\mathcal R} }_{0} < 1$, then s(M) < 0 and $s({J|}_{{{\mathscr{K}}}_{0}}) < 0$, the disease-free equilibrium ${{\mathscr{K}}}_{0}$ of system (1) is locally asymptotically stable.

## Analysis of Backward Bifurcation

In this subsection, we analyze the existence of Backward bifurcation for the model (1). To analyze this, we use the center manifold theory as described in Castillo-Chavez and Song (2004) (Theorem 4.1)^35^, which is reproduced here below for convenience.

**Theorem 0.2**. (*Theorem 4*.*1 of Castillo*-*Chavez and Song* (*2004*))^35^ *Consider the following general system of ordinary differential equations with a parameter ϕ*.

3 $$\frac{dx}{dt}=f(x,\varphi ),\,f:{{\mathbb{R}}}^{n}\times {\mathbb{R}}\to {\mathbb{R}}\,and\,f\in {{\mathbb{C}}}^{2}({{\mathbb{R}}}^{n}\times {\mathbb{R}})$$

*where* 0 *is an equilibrium point of the system* (*that is*, $f\mathrm{(0},\varphi )=0,\forall \,\varphi$) *and assume*

- $A={D}_{x}f\mathrm{(0},\mathrm{0)}=(\tfrac{\partial {f}_{i}}{{\partial }_{{x}_{j}}}\mathrm{(0,}\,\mathrm{0)})$ *is the linearization matrix of* (*3*) *around the equilibrium point 0 with ϕ evaluated at 0*. *Zero is a simple eigenvalue of A and other eigenvalues of A have negative real parts*;
- *Matrix A has a right eigenvector w and a left vector v* (*each corresponding to the zero eigenvalue*).

*Let f*_*k*_ *be the k*-*th component of f and*

$$a=\sum _{k,i,j=1}^{n}\,{v}_{k}{w}_{i}{w}_{j}\frac{{\partial }^{2}{f}_{k}}{\partial {x}_{i}\partial {x}_{j}}(0,0),$$

$$b=\sum _{k,i=1}^{n}\,{v}_{k}{w}_{i}\frac{{\partial }^{2}{f}_{k}}{\partial {x}_{i}\partial \varphi }(0,0).$$

*The local dynamics of the system (3) around 0 is totally determined by the signs of a and b*.

(i) *a* > 0, *b* > 0. *When ϕ* < 0 *with* $|\varphi |\ll 1$, *0 is locally asymptotically stable*, *and there exists a positive unstable equilibrium*; *when* $0 < \varphi \ll 1$, *0 is unstable and there exists a negative and locally asymptotically stable equilibrium*;
(ii) *a* < 0, *b* < 0. *When ϕ* < 0 *with* $|\varphi |\ll 1$, *0 is unstable*; *when* $0 < \varphi \ll 1$, *0 is locally asymptotically stable*, *and there exists a positive unstable equilibrium*;
(iii) *a* > 0, *b* < 0. *When ϕ* < 0 *with* $|\varphi |\ll 1$ *is unstable*, *and there exists a locally asymptotically stable negative equilibrium*; *when* $0 < \varphi \ll 1$, *0 is stable*, *and a positive unstable equilibrium appears*;
(iv) *a* < 0, *b* > 0. *When ϕ changes from negative to positive*, *0 changes its stability from stable to unstable*. *Correspondingly a negative unstable equilibrium becomes positive and locally asymptotically stable*.

Particularly, if *a* > 0 and *b* > 0, then a backward bifurcation occurs at *ϕ* = 0. If we choose *κ*_1_ as a bifurcation parameter, then at ${ {\mathcal R} }_{0}=1$, we have

$${\kappa }_{1}={\kappa }_{1}^{\ast }=\frac{{d}_{1}{d}_{2}^{2}(\gamma +{d}_{1})(\delta +{d}_{1})({d}_{2}+\mu )-\alpha \delta \eta {\kappa }_{2}\mu \varphi {{\rm{\Lambda }}}_{H}{{\rm{\Lambda }}}_{V}}{\delta \eta \mu \psi {{\rm{\Lambda }}}_{H}{{\rm{\Lambda }}}_{V}}.$$

Then, we make the following change of variables *S*_*H*_ = *x*_1_, *E*_*H*_ = *x*_2_, *I*_*H*_ = *x*_3_, *S*_*V*_ = *x*_4_, *E*_*V*_ = *x*_5_ and *I*_*V*_ = *x*_6_. In addition, using vector notation ${\bf{x}}={({x}_{1},{x}_{2},{x}_{3},{x}_{4},{x}_{5},{x}_{6})}^{T}$, the PWD model can then be written in the form $dx/dt=F({\bf{x}})$, with $F={({f}_{1},{f}_{2},{f}_{3},{f}_{4},{f}_{5},{f}_{6})}^{T}$, as shown below:

$$\{\begin{array}{rcl}\frac{d{x}_{1}}{dt} & = & {{\rm{\Lambda }}}_{H}-{\kappa }_{1}\psi {x}_{1}{x}_{6}-{\kappa }_{2}\varphi \alpha {x}_{1}{x}_{6}-{d}_{1}{x}_{1}={f}_{1},\\ \frac{d{x}_{2}}{dt} & = & {\kappa }_{1}\psi {x}_{1}{x}_{6}+{\kappa }_{2}\varphi \alpha {x}_{1}{x}_{6}-({d}_{1}+\delta ){x}_{2}={f}_{2}\\ \frac{d{x}_{3}}{dt} & = & \delta {x}_{2}-({d}_{1}+\gamma ){x}_{3}={f}_{3}\\ \frac{d{x}_{4}}{dt} & = & {{\rm{\Lambda }}}_{V}-\eta {x}_{4}{x}_{3}-{d}_{2}{x}_{4}={f}_{4}\\ \frac{d{x}_{5}}{dt} & = & \eta {x}_{4}{x}_{3}-({d}_{2}+\mu ){x}_{5}={f}_{5}\\ \frac{d{x}_{6}}{dt} & = & \mu {x}_{5}-{d}_{2}{x}_{6}={f}_{6}\mathrm{.}\end{array}$$

To follow the above method, we find the Jacobian matrix evaluated at disease free equilibrium ${{\mathscr{K}}}_{0}$ is given by

$$J({{\mathscr{K}}}_{0})=(\begin{array}{cccccc}-{d}_{1} & 0 & 0 & 0 & 0 & -\tfrac{(\gamma +{d}_{1})(\delta +{d}_{1}){d}_{2}^{2}(\mu +{d}_{2})}{\delta \eta \mu {{\rm{\Lambda }}}_{V}}\\ 0 & -(\delta +{d}_{1}) & 0 & 0 & 0 & \tfrac{(\gamma +{d}_{1})(\delta +{d}_{1}){d}_{2}^{2}(\mu +{d}_{2})}{\delta \eta \mu {{\rm{\Lambda }}}_{V}}\\ 0 & \delta  & -(\gamma +{d}_{1}) & 0 & 0 & 0\\ 0 & 0 & -\tfrac{\eta {{\rm{\Lambda }}}_{V}}{{d}_{2}} & -{d}_{2} & 0 & 0\\ 0 & 0 & \tfrac{\eta {{\rm{\Lambda }}}_{V}}{{d}_{2}} & 0 & -(\mu +{d}_{2}) & 0\\ 0 & 0 & 0 & 0 & \mu  & -{d}_{2}\end{array})$$

The Jacobian matrix $J({{\mathscr{K}}}_{0})$ has a simple zero eigenvalue evaluated at ${\kappa }_{1}^{\ast }$. The Jacobian matrix $J({{\mathscr{K}}}_{0})$ evaluated at ${\kappa }_{1}^{\ast }$ has right and left eigenvector denoted by ${\bf{W}}={[{w}_{1},{w}_{2},{w}_{3},{w}_{4},{w}_{5},{w}_{6}]}^{T}$ and ${\bf{V}}=[{v}_{1},{v}_{2},{v}_{3},{v}_{4},{v}_{5},{v}_{6}]$ respectively, and obtain

$$\begin{array}{l}{w}_{1}=-\frac{{w}_{2}(\delta +{d}_{1})}{{d}_{1}},\,{w}_{2} > \mathrm{0,}\,{w}_{3}=\frac{\delta {w}_{2}}{\gamma +{d}_{1}},\,{w}_{4}=-\frac{\delta \eta {w}_{2}{{\rm{\Lambda }}}_{V}}{{d}_{2}^{2}(\gamma +{d}_{1})},\\ {w}_{5}=\frac{\delta \eta {w}_{2}{{\rm{\Lambda }}}_{V}}{{d}_{2}(\gamma +{d}_{1})({d}_{2}+\mu )},\,{w}_{6}=\frac{\delta \eta \mu {w}_{2}{{\rm{\Lambda }}}_{V}}{{d}_{2}^{2}(\gamma +{d}_{1})({d}_{2}+\mu )}\end{array}$$

and

$$\begin{array}{l}{v}_{1}={v}_{4}=\mathrm{0,}\,{v}_{2} > 0,\,{v}_{3}=\frac{{v}_{2}(\delta +{d}_{1})}{\delta },\\ {v}_{5}=\frac{{d}_{2}{v}_{2}(\gamma +{d}_{1})(\delta +{d}_{1})}{\delta \eta {{\rm{\Lambda }}}_{V}},\,{v}_{6}=\frac{{d}_{2}{v}_{2}(\gamma +{d}_{1})(\delta +{d}_{1})({d}_{2}+\mu )}{\delta \eta \mu {{\rm{\Lambda }}}_{V}}.\end{array}$$

**Computation of a:**

To compute *a*, we find the following partial derivatives

$$\frac{{\partial }^{2}{f}_{2}}{\partial {x}_{1}\partial {x}_{6}}=\frac{{\partial }^{2}{f}_{2}}{\partial {x}_{1}\partial {x}_{6}}={\kappa }_{1}\psi ,\frac{{\partial }^{2}{f}_{2}}{\partial {x}_{1}\partial {x}_{6}}=\frac{{\partial }^{2}{f}_{2}}{\partial {x}_{1}\partial {x}_{6}}={\kappa }_{1}\varphi \alpha ,\frac{{\partial }^{2}{f}_{5}}{\partial {x}_{3}\partial {x}_{4}}=\frac{{\partial }^{2}{f}_{5}}{\partial {x}_{3}\partial {x}_{4}}=\eta .$$

we obtain

$$a=-\frac{2\delta \eta {w}_{2}^{2}{{\rm{\Lambda }}}_{V}({\kappa }_{1}\mu {v}_{2}(\gamma +{d}_{1})(\delta +{d}_{1})(\psi +\varphi \alpha )+\delta {d}_{1}\eta {v}_{5}({d}_{2}+\mu ))}{{d}_{1}{d}_{2}^{2}{(\gamma +{d}_{1})}^{2}({d}_{2}+\mu )}.$$

**Computation of b:**

To compute *b*, we find the following partial derivatives

$$\frac{{\partial }^{2}{f}_{2}}{\partial {x}_{6}\partial {\kappa }_{1}}=\frac{\psi {{\rm{\Lambda }}}_{H}}{{d}_{1}}$$

we obtain

$$b=\frac{\psi \delta \eta \mu {v}_{2}{w}_{2}{{\rm{\Lambda }}}_{H}{{\rm{\Lambda }}}_{V}}{{d}_{1}{d}_{2}^{2}(\gamma +{d}_{1})({d}_{2}+\mu )}$$

Obviously, the coefficient *b* is positive always so that, according to Theorem (0.2), it is the sign of the coefficient *a* which decides the local dynamics around the disease-free equilibrium for ${\kappa }_{1}={\kappa }_{1}^{\ast }$.

**Theorem 0.3**. If ${ {\mathcal R} }_{0} < 1$, then the disease-free equilibrium ${{\mathscr{K}}}_{0}$ is globally asymptotically stable on ${\rm{\Psi }}$.

**Proof:** Consider the following lyapunov function:

$$L={w}_{1}{\int }_{{S}_{H}^{0}}^{{S}_{H}}\,(1-\frac{{S}_{H}^{0}}{y})dy+{w}_{2}{E}_{H}+{w}_{3}{I}_{H}+{w}_{4}{\int }_{{S}_{V}^{0}}^{{S}_{V}}\,(1-\frac{{S}_{V}^{0}}{y})dy+{w}_{5}{E}_{V}+{w}_{6}{I}_{V}.$$

The derivative of *L* along the solution of model (1) is

$$L^{\prime} ={w}_{1}(1-\frac{{S}_{H}^{0}}{{S}_{H}}){S}_{H}^{^{\prime} }+{w}_{2}{E}_{H}^{^{\prime} }+{w}_{3}{I}_{H}^{^{\prime} }+{w}_{4}(1-\frac{{S}_{V}^{0}}{{S}_{V}}){S}_{V}^{^{\prime} }+{w}_{5}{E}_{V}^{^{\prime} }+{w}_{6}{I}_{V}^{^{\prime} }\mathrm{.}$$

where *w*_*i*_, for $i=1,2,\ldots 6$ are positive constants to be chosen later.

4 $$\begin{array}{rcl}L^{\prime}  & = & {w}_{1}(1-\frac{{S}_{H}^{0}}{{S}_{H}})[{{\rm{\Lambda }}}_{H}-{\kappa }_{1}\psi {S}_{H}{I}_{V}-{\kappa }_{2}\varphi \alpha {S}_{H}{I}_{V}-{d}_{1}{S}_{H}]\\  &  & +\,{w}_{2}[{\kappa }_{1}\psi {S}_{H}{I}_{V}+{\kappa }_{2}\varphi \alpha {S}_{H}{I}_{V}-({d}_{1}+\delta ){E}_{H}]\\  &  & +\,{w}_{3}[\delta {E}_{H}-({d}_{1}+\gamma ){I}_{H}]+{w}_{4}(1-\frac{{S}_{V}^{0}}{{S}_{V}})[{{\rm{\Lambda }}}_{V}-\eta {S}_{V}{I}_{H}-{d}_{2}{S}_{V}]\\  &  & +\,{w}_{5}[\eta {S}_{V}{I}_{H}-({d}_{2}+\mu ){E}_{V}]+{w}_{6}[\mu {E}_{V}-{d}_{2}{I}_{V}]\end{array}$$

Using ${S}_{H}^{0}=\frac{{{\rm{\Lambda }}}_{H}}{{d}_{1}}$ and ${S}_{V}^{0}=\frac{{{\rm{\Lambda }}}_{V}}{{d}_{2}}$ in equation (6), we get

5 $$\begin{array}{rcl}L^{\prime}  & = & -{d}_{1}{w}_{1}\tfrac{{({S}_{H}-{S}_{H}^{0})}^{2}}{{S}_{H}}-{d}_{2}{w}_{4}\tfrac{{({S}_{V}-{S}_{V}^{0})}^{2}}{{S}_{V}}+({w}_{2}-{w}_{1})[{\kappa }_{1}\psi {S}_{H}{I}_{V}+{\kappa }_{2}\varphi \alpha {S}_{H}{I}_{V}]\\  &  & +\,[{w}_{1}\tfrac{{{\rm{\Lambda }}}_{H}}{{d}_{1}}({\kappa }_{1}\psi +{\kappa }_{2}\varphi \alpha )-{d}_{2}{w}_{6}]\,{I}_{V}+[{w}_{3}\delta -{w}_{2}({d}_{1}+\delta )]{E}_{H}\\  &  & +\,({w}_{5}-{w}_{4})\eta {S}_{V}{I}_{H}+[{w}_{4}\eta \tfrac{{{\rm{\Lambda }}}_{V}}{{d}_{2}}-{w}_{3}({d}_{1}+\gamma )]{I}_{H}\\  &  & +\,[{w}_{6}\mu -{w}_{5}({d}_{2}+\mu )]{E}_{V}\mathrm{.}\end{array}$$

Now choosing the constants such as *w*_1_ = *w*_2_ = *δ*, *w*_3_ = (*d*_1_ + *δ*), ${w}_{4}={w}_{5}=\frac{{d}_{2}({d}_{1}+\gamma )({d}_{1}+\delta )}{\eta {{\rm{\Lambda }}}_{V}}$, ${w}_{6}=\frac{{d}_{2}({d}_{1}+\gamma )({d}_{2}+\mu )({d}_{1}+\delta )}{\eta \mu {{\rm{\Lambda }}}_{V}}$ and simplifying, we obtain

6 $$\begin{array}{rcl}L^{\prime}  & = & -{d}_{1}\delta \frac{{({S}_{H}-{S}_{H}^{0})}^{2}}{{S}_{H}}-\frac{{d}_{2}^{2}({d}_{1}+\gamma )({d}_{1}+\delta )}{\eta {{\rm{\Lambda }}}_{V}}\frac{{({S}_{V}-{S}_{V}^{0})}^{2}}{{S}_{V}}\\  &  & -\frac{{d}_{2}^{2}({d}_{1}+\gamma )({d}_{2}+\mu )({d}_{1}+\delta )}{\eta \mu {{\rm{\Lambda }}}_{V}}\mathrm{(1}-{ {\mathcal R} }_{0}^{2}){I}_{V}\mathrm{.}\end{array}$$

Thus, *L*′(*t*) is negative for ${ {\mathcal R} }_{0}\le 1$ and zero if and only if ${S}_{H}={S}_{H}^{0}$, ${S}_{V}={S}_{V}^{0}$, *E*_*H*_ = *I*_*H*_ = 0 and *E*_*V*_ = *I*_*V*_ = 0. Therefore the largest compact invariant set in ${\rm{\Psi }}$ is the singleton set ${{\mathscr{K}}}_{0}$. So, the model (1) is globally asymptotically stable.

## Stability Endemic Equilibrium

In this subsection, we investigate the stability results for the endemic case. The endemic equilibrium of the model (1) denoted by ${{\mathscr{K}}}_{1}=({S}_{H}^{\ast },{E}_{H}^{\ast },{I}_{H}^{\ast },{S}_{V}^{\ast },{E}_{V}^{\ast },{I}_{V}^{\ast })$ and is given by

$$\{\begin{array}{rcl}{S}_{H}^{\ast } & = & \frac{{d}_{2}({d}_{2}+\mu )({d}_{2}(\gamma +{d}_{1})(\delta +{d}_{1})+\delta \eta {{\rm{\Lambda }}}_{H})}{\delta \eta ({d}_{1}{d}_{2}({d}_{2}+\mu )+{{\rm{\Lambda }}}_{V}{\mu }_{V}(\alpha {\kappa }_{2}\varphi +{\kappa }_{1}\psi ))}\\ {E}_{H}^{\ast } & = & \frac{{d}_{1}{d}_{2}^{2}(\gamma +{d}_{1})(\delta +{d}_{1})({d}_{2}+\mu )({ {\mathcal R} }_{0}-\mathrm{1)}}{\delta \eta (\delta +{d}_{1})({d}_{1}{d}_{2}({d}_{2}+\mu )+{{\rm{\Lambda }}}_{V}\mu (\alpha {\kappa }_{2}\varphi +{\kappa }_{1}\psi ))}\\ {I}_{H}^{\ast } & = & \frac{{d}_{1}{d}_{2}^{2}(\gamma +{d}_{1})(\delta +{d}_{1})({d}_{2}+\mu )({ {\mathcal R} }_{0}-\mathrm{1)}}{\eta (\gamma +{d}_{1})(\delta +{d}_{1})({d}_{1}{d}_{2}({d}_{2}+\mu )+{{\rm{\Lambda }}}_{V}\mu (\alpha {\kappa }_{2}\varphi +{\kappa }_{1}\psi ))}\\ {S}_{V}^{\ast } & = & \frac{(\gamma +{d}_{1})(\delta +{d}_{1})({d}_{1}{d}_{2}({d}_{2}+\mu )+{{\rm{\Lambda }}}_{V}{\mu }_{V}(\alpha {\kappa }_{2}\varphi +{\kappa }_{1}\psi ))}{{\mu }_{V}(\alpha {\kappa }_{2}\varphi +{\kappa }_{1}\psi )({d}_{2}(\gamma +{d}_{1})(\delta +{d}_{1})+\delta \eta {{\rm{\Lambda }}}_{H})}\\ {E}_{V}^{\ast } & = & \frac{{d}_{1}{d}_{2}^{2}(\gamma +{d}_{1})(\delta +{d}_{1})({d}_{2}+\mu )({ {\mathcal R} }_{0}-\mathrm{1)}}{({d}_{2}+\mu )\mu (\alpha {\kappa }_{2}\varphi +{\kappa }_{1}\psi )({d}_{2}(\gamma +{d}_{1})(\delta +{d}_{1})+\delta \eta {{\rm{\Lambda }}}_{H})}\\ {I}_{V}^{\ast } & = & \frac{{d}_{1}{d}_{2}^{2}(\gamma +{d}_{1})(\delta +{d}_{1})({d}_{2}+\mu )({ {\mathcal R} }_{0}-\mathrm{1)}}{{d}_{2}({d}_{2}+\mu )(\alpha {\kappa }_{2}\varphi +{\kappa }_{1}\psi )({d}_{2}(\gamma +{d}_{1})(\delta +{d}_{1})+\delta \eta {{\rm{\Lambda }}}_{H})}\end{array}$$

**Lemma 0.1**. *A unique endemic equilibrium exists for model* (*1*) *whenever* ${ {\mathcal R} }_{0} > 1$.

**Theorem 0.4**. *The endemic equilibrium of the model* (*1*) *is locally asymptotically stable when* ${ {\mathcal R} }_{0} > 1$ *and the conditions*

- $${c}_{1}{c}_{2}-{c}_{3} > 0,$$
- $${c}_{3}({c}_{1}{c}_{2}-{c}_{3})+{c}_{1}({c}_{5}-{c}_{1}{c}_{4}) > 0,$$
- $$({c}_{1}{c}_{2}-{c}_{3})({c}_{3}{c}_{4}-{c}_{2}{c}_{5})-{({c}_{5}-{c}_{1}{c}_{4})}^{2} > 0$$.

*are satisfied*.

**Proof:** The jacobian matrix evaluated at ${{\mathscr{K}}}_{1}$ is

$${J}_{{{\mathscr{K}}}_{1}}=(\begin{array}{cccccc}-({d}_{1}+P{I}_{V}^{\ast }) & 0 & 0 & 0 & 0 & -P{S}_{H}^{\ast }\\ P{I}_{V}^{\ast } & -{Q}_{1} & 0 & 0 & 0 & P{S}_{H}^{\ast }\\ 0 & \delta  & -{Q}_{2} & 0 & 0 & 0\\ 0 & 0 & -\eta {S}_{V}^{\ast } & -({d}_{2}+\eta {I}_{H}^{\ast }) & 0 & 0\\ 0 & 0 & \eta {S}_{V}^{\ast } & \eta {I}_{H}^{\ast } & -{Q}_{3} & 0\\ 0 & 0 & 0 & 0 & {\mu }_{V} & -{d}_{2}\end{array}).$$

The associated characteristics equation of ${J}_{{{\mathscr{K}}}_{0}}$ is

7 $$(\lambda +{d}_{2})[{\lambda }^{5}+{c}_{1}{\lambda }^{4}+{c}_{2}{\lambda }^{3}+{c}_{3}{\lambda }^{2}+{c}_{4}\lambda +{c}_{5}]=0,$$

where

$$\begin{array}{rcl}{c}_{1} & = & {d}_{1}+{d}_{2}+\eta {I}_{H}^{\ast }+P{I}_{V}^{\ast }+{Q}_{1}+{Q}_{2}+{Q}_{3},\\ {c}_{2} & = & {d}_{1}({d}_{2}+\eta {I}_{H}^{\ast }+{Q}_{1}+{Q}_{2}+{Q}_{3})+{d}_{2}(P{I}_{V}^{\ast }+{Q}_{1}+{Q}_{2}+{Q}_{3})\\  &  & +\,{Q}_{3}(\eta {I}_{H}^{\ast }+P{I}_{V}^{\ast }+{Q}_{1}+{Q}_{2})+\eta P{I}_{H}^{\ast }{I}_{V}^{\ast }+\eta {Q}_{1}{I}_{H}^{\ast }+\eta {Q}_{2}{I}_{H}^{\ast }\\  &  & +\,P{Q}_{1}{I}_{V}^{\ast }+P{Q}_{2}{I}_{V}^{\ast }+{Q}_{1}{Q}_{2},\\ {c}_{3} & = & {d}_{1}({d}_{2}({Q}_{1}+{Q}_{2}+{Q}_{3})+\eta ({Q}_{1}+{Q}_{2}+{Q}_{3}){I}_{H}^{\ast }+{Q}_{1}{Q}_{2}+({Q}_{1}+{Q}_{2}){Q}_{3})\\  &  & +\,{d}_{2}(P({Q}_{1}+{Q}_{2}+{Q}_{3}){I}_{V}^{\ast }+{Q}_{2}{Q}_{3}+{Q}_{1}({Q}_{2}+{Q}_{3}))\\  &  & +\,\eta P{Q}_{1}{I}_{H}^{\ast }{I}_{V}^{\ast }+\eta P{Q}_{2}{I}_{H}^{\ast }{I}_{V}^{\ast }+{Q}_{3}(\eta {I}_{H}^{\ast }(P{I}_{V}^{\ast }+{Q}_{1}+{Q}_{2})\\  &  & +\,P({Q}_{1}+{Q}_{2}){I}_{V}^{\ast }+{Q}_{1}{Q}_{2})+\eta {Q}_{2}{Q}_{1}{I}_{H}^{\ast }+P{Q}_{2}{Q}_{1}{I}_{V}^{\ast },\\ {c}_{4} & = & {Q}_{1}{Q}_{2}({d}_{2}+\eta {I}_{H}^{\ast })({d}_{1}+P{I}_{V}^{\ast })+{Q}_{3}({Q}_{1}({d}_{2}+\eta {I}_{H}^{\ast })({d}_{1}+P{I}_{V}^{\ast })\\  &  & +\,{Q}_{2}({Q}_{1}({d}_{1}+{d}_{2}+\eta {I}_{H}^{\ast }+P{I}_{V}^{\ast })+({d}_{2}+\eta {I}_{H}^{\ast })({d}_{1}+P{I}_{V}^{\ast }))),\\ {c}_{5} & = & {Q}_{1}{Q}_{2}{Q}_{3}({d}_{2}+\eta {I}_{H}^{\ast })({d}_{1}+P{I}_{V}^{\ast })+\delta \eta {P}^{2}{S}_{H}^{\ast }{I}_{V}^{\ast }{S}_{V}^{\ast }{\mu }_{V}\mathrm{.}\end{array}$$

and $(\delta +{d}_{1})={Q}_{1},\,(\gamma +{d}_{1})={Q}_{2},\,({d}_{2}+\mu )={Q}_{3}$ and $P=({\kappa }_{1}\psi +{\kappa }_{2}\varphi \alpha )$. In equation (7) one of the root −*d*_2_ is clearly negative, for the remaining the roots we use the following Routh-Hurtwiz conditions. *c*_*i*_ > 0 for $i=1,2,\ldots 5$ where

8 $$\begin{array}{l}{H}_{1}={c}_{1},\,{H}_{2}=(\begin{array}{cc}{c}_{1} & 1\\ {c}_{3} & {c}_{2}\end{array}),\,{H}_{3}=(\begin{array}{ccc}{c}_{1} & 1 & 0\\ {c}_{3} & {c}_{2} & {c}_{1}\\ {c}_{5} & {c}_{4} & {c}_{3}\end{array}),\\ {H}_{4}=(\begin{array}{cccc}{c}_{1} & 1 & 0 & 0\\ {c}_{3} & {c}_{2} & 1 & 0\\ {c}_{5} & {c}_{4} & {c}_{3} & {c}_{2}\\ 0 & 0 & {c}_{5} & {c}_{4}\end{array}),\,{H}_{5}=(\begin{array}{ccccc}{c}_{1} & 1 & 0 & 0 & 0\\ {c}_{3} & {c}_{2} & {c}_{1} & 1 & 0\\ {c}_{5} & {c}_{4} & {c}_{3} & {c}_{2} & {c}_{1}\\ 0 & 0 & {c}_{5} & {c}_{4} & {c}_{3}\\ 0 & 0 & 0 & 0 & {c}_{5}\end{array}).\end{array}$$

Here, all *c*_*i*_ > 0 for $i=1,2,\ldots 5$ and the parameters are positive. Thus, the given conditions in the theorem above ensure the local endemic stability of the endemic equilibrium of the model (1).

## Global stability of Endemic Equilibrium

Here, we present the global stability of the system (1) at ${{\mathscr{K}}}_{1}$ by using the approach presented in refs 36–38. At the steady state the model (1) at ${{\mathscr{K}}}_{1}$ is

9 $$\begin{array}{rcl}{{\rm{\Lambda }}}_{H} & = & P{S}_{H}^{\ast }{I}_{V}^{\ast }+{d}_{1}{S}_{H}^{\ast },\,({d}_{1}+\delta ){E}_{H}^{\ast }=P{S}_{H}^{\ast }{I}_{V}^{\ast },\,\delta {E}_{H}^{\ast }=({d}_{1}+\gamma ){I}_{H}^{\ast }\\ \frac{({d}_{1}+\gamma )({d}_{1}+\delta )}{\delta }{I}_{H}^{\ast } & = & P{S}_{H}^{\ast }{I}_{V}^{\ast },{{\rm{\Lambda }}}_{V}=\eta {S}_{V}^{\ast }{I}_{H}^{\ast }+{d}_{2}{S}_{V}^{\ast },\eta {S}_{V}^{\ast }{I}_{H}^{\ast }=({d}_{2}+\mu ){E}_{V}^{\ast },\\ \mu {E}_{V}^{\ast } & = & {d}_{2}{I}_{V}^{\ast },\eta {S}_{V}^{\ast }{I}_{H}^{\ast }=\frac{({d}_{2}+\mu ){d}_{2}{I}_{V}^{\ast }}{\mu }.\end{array}$$

The above equations will be used in the following equations (10–15).

**Theorem 0.5**. *If* ${ {\mathcal R} }_{0} > 1$, *then the endemic equilibrium* ${{\mathscr{K}}}_{1}$ *is globally asymptotically stable*.

**Proof:** Consider the lyapunove function:

$$\begin{array}{rcl}L & = & {\int }_{{S}_{H}^{\ast }}^{{S}_{H}}\,(1-\frac{{S}_{H}^{\ast }}{x})dx+{\int }_{{E}_{H}^{\ast }}^{{E}_{H}}\,(1-\frac{{E}_{H}^{\ast }}{x})dx+\frac{({d}_{1}+\delta )}{\delta }{\int }_{{I}_{H}^{\ast }}^{{I}_{H}}\,(1-\frac{{I}_{H}^{\ast }}{x})dx\\  &  & +{\int }_{{S}_{V}^{\ast }}^{{S}_{V}}\,(1-\frac{{S}_{V}^{\ast }}{x})dx+{\int }_{{E}_{V}^{\ast }}^{{E}_{V}}\,(1-\frac{{E}_{V}^{\ast }}{x})dx+\frac{({d}_{2}+\mu )}{\mu }{\int }_{{I}_{V}^{\ast }}^{{I}_{V}}\,(1-\frac{{I}_{V}^{\ast }}{x})dx\mathrm{.}\end{array}$$

The derivative of *L* along the solutions of system (1) is

$$\begin{array}{rcl}\dot{L} & = & (1-\frac{{S}_{H}^{\ast }}{{S}_{H}}){S}_{H}^{^{\prime} }+(1-\frac{{E}_{H}^{\ast }}{{E}_{H}}){E}_{H}^{^{\prime} }+\frac{({d}_{1}+\delta )}{\delta }(1-\frac{{I}_{H}^{\ast }}{{I}_{H}}){I}_{H}^{^{\prime} }\\  &  & +\,(1-\frac{{S}_{V}^{\ast }}{{S}_{V}}){S}_{V}^{^{\prime} }+(1-\frac{{E}_{V}^{\ast }}{{E}_{V}}){E}_{V}^{^{\prime} }+\frac{({d}_{2}+\mu )}{\mu }(1-\frac{{I}_{V}^{\ast }}{{I}_{V}}){I}_{V}^{^{\prime} }.\end{array}$$

By direct calculations, we have that:

10 $$\begin{array}{rcl}(1-\frac{{S}_{H}^{\ast }}{{S}_{H}}){S}_{H}^{^{\prime} } & = & (1-\frac{{S}_{H}^{\ast }}{{S}_{H}})[{{\rm{\Lambda }}}_{H}-P{S}_{H}{I}_{V}-{d}_{1}{S}_{H}]\\  & = & (1-\frac{{S}_{H}^{\ast }}{{S}_{H}})[P{S}_{H}^{\ast }{I}_{V}^{\ast }+{d}_{1}{S}_{H}^{\ast }-P{S}_{H}{I}_{V}-{d}_{1}{S}_{H}]\\  & = & {d}_{1}{S}_{H}^{\ast }(2-\frac{{S}_{H}}{{S}_{H}^{\ast }}-\frac{{S}_{H}^{\ast }}{{S}_{H}})+(1-\frac{{S}_{H}^{\ast }}{{S}_{H}})P{S}_{H}^{\ast }{I}_{V}^{\ast }-P{S}_{H}{I}_{V}(1-\frac{{S}_{H}^{\ast }}{{S}_{H}})\\  & = & {d}_{1}{S}_{H}^{\ast }(2-\frac{{S}_{H}}{{S}_{H}^{\ast }}-\frac{{S}_{H}^{\ast }}{{S}_{H}})+(1-\frac{{S}_{H}^{\ast }}{{S}_{H}})P{S}_{H}^{\ast }{I}_{V}^{\ast }-P{S}_{H}{I}_{V}+P{S}_{H}^{\ast }{I}_{V},\end{array}$$

11 $$\begin{array}{rcl}(1-\frac{{E}_{H}^{\ast }}{{E}_{H}}){E}_{H}^{^{\prime} } & = & (1-\frac{{E}_{H}^{\ast }}{{E}_{H}})[P{S}_{H}{I}_{V}-({d}_{1}+\delta ){E}_{H}]\\  & = & P{S}_{H}{I}_{V}-P{S}_{H}{I}_{V}\frac{{E}_{H}^{\ast }}{{E}_{H}}-({d}_{1}+\delta ){E}_{H}+({d}_{1}+\delta ){E}_{H}^{\ast }\\  & = & P{S}_{H}{I}_{V}-P{S}_{H}{I}_{V}\frac{{E}_{H}^{\ast }}{{E}_{H}}-({d}_{1}+\delta ){E}_{H}+P{S}_{H}^{\ast }{I}_{V}^{\ast },\end{array}$$

12 $$\begin{array}{rcl}(1-\frac{{I}_{H}^{\ast }}{{I}_{H}})\frac{({d}_{1}+\delta )}{\delta }{I}_{H}^{^{\prime} } & = & (1-\frac{{I}_{H}^{\ast }}{{I}_{H}})\frac{({d}_{1}+\delta )}{\delta }[\delta {E}_{H}-({d}_{1}+\gamma ){I}_{H}]\\  & = & ({d}_{1}+\delta ){E}_{H}-({d}_{1}+\delta ){E}_{H}\frac{{I}_{H}^{\ast }}{{I}_{H}}\\  &  & -\,\frac{({d}_{1}+\delta )({d}_{1}+\gamma )}{\delta }{I}_{H}+\frac{({d}_{1}+\delta )({d}_{1}+\gamma )}{\delta }{I}_{H}^{\ast },\end{array}$$

13 $$\begin{array}{rcl}(1-\frac{{S}_{V}^{\ast }}{{S}_{V}}){S}_{V}^{^{\prime} } & = & (1-\frac{{S}_{V}^{\ast }}{{S}_{V}})[{{\rm{\Lambda }}}_{V}-\eta {S}_{V}{I}_{H}-{d}_{2}{S}_{V}]\\  & = & (1-\frac{{S}_{V}^{\ast }}{{S}_{V}})[\eta {S}_{V}^{\ast }{I}_{H}^{\ast }+{d}_{2}{S}_{V}^{\ast }-\eta {S}_{V}{I}_{H}-{d}_{2}{S}_{V}]\\  & = & {d}_{2}{S}_{V}^{\ast }(2-\frac{{S}_{V}}{{S}_{V}^{\ast }}-\frac{{S}_{V}^{\ast }}{{S}_{V}})+(1-\frac{{S}_{V}^{\ast }}{{S}_{V}})\eta {S}_{V}^{\ast }{I}_{H}^{\ast }-\eta {S}_{V}{I}_{H}+\eta {S}_{V}^{\ast }{I}_{H},\end{array}$$

14 $$\begin{array}{rcl}(1-\frac{{E}_{V}^{\ast }}{{E}_{V}}){E}_{V}^{^{\prime} } & = & (1-\frac{{E}_{V}^{\ast }}{{E}_{V}})[\eta {S}_{V}{I}_{H}-({d}_{2}+\mu ){E}_{V}]\\  & = & \eta {S}_{V}{I}_{H}-\eta {S}_{V}{I}_{H}\frac{{E}_{V}^{\ast }}{{E}_{V}}-({d}_{2}+\mu ){E}_{V}+({d}_{2}+\mu ){E}_{V}^{\ast }\\  & = & \eta {S}_{V}{I}_{H}-\eta {S}_{V}{I}_{H}\frac{{E}_{V}^{\ast }}{{E}_{V}}-({d}_{2}+\mu ){E}_{V}+\eta {S}_{V}^{\ast }{I}_{H}^{\ast },\end{array}$$

and

15 $$\begin{array}{rcl}(1-\frac{{I}_{V}^{\ast }}{{I}_{V}})\frac{({d}_{2}+\mu )}{\mu }{I}_{V}^{^{\prime} } & = & (1-\frac{{I}_{V}^{\ast }}{{I}_{V}})\frac{({d}_{2}+\mu )}{\mu }[\mu {E}_{V}-{d}_{2}{I}_{V}]\\  & = & ({d}_{2}+\mu ){E}_{V}-({d}_{2}+\mu ){E}_{V}\frac{{I}_{V}^{\ast }}{{I}_{V}}-\frac{({d}_{2}+\mu ){d}_{2}}{\mu }{I}_{V}+\frac{({d}_{2}+\mu ){d}_{2}}{\mu }{I}_{V}^{\ast }\\  & = & ({d}_{2}+\mu ){E}_{V}-\frac{\eta {S}_{V}^{\ast }{I}_{H}^{\ast }}{{E}_{V}^{\ast }}{E}_{V}\frac{{I}_{V}^{\ast }}{{I}_{V}}-\frac{\eta {S}_{V}^{\ast }{I}_{H}^{\ast }}{{I}_{V}^{\ast }}{I}_{V}+\eta {S}_{V}^{\ast }{I}_{H}^{\ast }.\end{array}$$

It follows from (10–15)

16 $$\begin{array}{rcl}\dot{L} & = & {d}_{1}{S}_{H}^{\ast }(2-\frac{{S}_{H}}{{S}_{H}^{\ast }}-\frac{{S}_{H}^{\ast }}{{S}_{H}})+P{S}_{H}^{\ast }{I}_{V}^{\ast }(3-\frac{{S}_{H}^{\ast }}{{S}_{H}}-\frac{{I}_{H}}{{I}_{H}^{\ast }}-\frac{{I}_{H}^{\ast }{E}_{H}}{{E}_{H}^{\ast }{I}_{H}}-\frac{{I}_{V}}{{I}_{V}^{\ast }}(\frac{{S}_{H}{E}_{H}^{\ast }}{{S}_{H}^{\ast }{E}_{H}}-1))\\  &  & +{d}_{2}{S}_{V}^{\ast }(2-\frac{{S}_{V}}{{S}_{V}^{\ast }}-\frac{{S}_{V}^{\ast }}{{S}_{V}})+\eta {S}_{V}^{\ast }{I}_{H}^{\ast }(3-\frac{{S}_{V}^{\ast }}{{S}_{V}}-\frac{{I}_{V}}{{I}_{V}^{\ast }}-\frac{{E}_{V}{I}_{V}^{\ast }}{{E}_{V}^{\ast }{I}_{V}}-\frac{{I}_{H}}{{I}_{H}^{\ast }}(\frac{{S}_{V}{E}_{V}^{\ast }}{{E}_{V}{S}_{V}^{\ast }}-1))\end{array}$$

In equation (16),

$$\begin{array}{rcl}{d}_{1}{S}_{H}^{\ast }(2-\frac{{S}_{H}}{{S}_{H}^{\ast }}-\frac{{S}_{H}^{\ast }}{{S}_{H}}) & \le  & 0,\\ {d}_{2}{S}_{V}^{\ast }(2-\frac{{S}_{V}}{{S}_{V}^{\ast }}-\frac{{S}_{V}^{\ast }}{{S}_{V}}) & \le  & 0,\\ (3-\frac{{S}_{H}^{\ast }}{{S}_{H}}-\frac{{I}_{H}}{{I}_{H}^{\ast }}-\frac{{I}_{H}^{\ast }{E}_{H}}{{E}_{H}^{\ast }{I}_{H}}-\frac{{I}_{V}}{{I}_{V}^{\ast }}(\frac{{S}_{H}{E}_{H}^{\ast }}{{S}_{H}^{\ast }{E}_{H}}-1)) & \le  & 0,\\ (3-\frac{{S}_{V}^{\ast }}{{S}_{V}}-\frac{{I}_{V}}{{I}_{V}^{\ast }}-\frac{{E}_{V}{I}_{V}^{\ast }}{{E}_{V}^{\ast }{I}_{V}}-\frac{{I}_{H}}{{I}_{H}^{\ast }}(\frac{{S}_{V}{E}_{V}^{\ast }}{{E}_{V}{S}_{V}^{\ast }}-1)) & \le  & 0.\end{array}$$

One can see that the largest invariant subset, $\dot{L}=0$ is ${{\mathscr{K}}}_{1}$. So, by LaSalle’s invariance Principle^39^, ${{\mathscr{K}}}_{1}$ is globally asymptotically stable whenever ${ {\mathcal R} }_{0} > 1$.

## Optimal Control Problem

Here, we incorporate control measures into the system (1) by including the density effects respectively to modifying the recruitment rate of the susceptible classes, that is, ${{\rm{\Lambda }}}_{H}\to {{\rm{\Lambda }}}_{H}+c{N}_{H}$ and ${{\rm{\Lambda }}}_{V}\to {{\rm{\Lambda }}}_{V}{N}_{V}$, where the constant *c* represent the density impact on recruitment rate^40^. Our main goal is to investigate the best control strategies with minimum cost of implementation. The force of infections in pine trees population is reduce by (1 − *u*_1_), where *u*_1_ measures the effort due to the precautionary measures, such as nematode tree-injection and vaccination. In order to prevent infection of the population of pine trees, a nematicide-injection preventative control is used. The deforestation of infected trees effort is denoted by the control variable *u*_2_. The destruction and removal of infected pine trees can drastically reduce further infection. This will further ensure that eggs, larvae and pupa that are inhabiting the pines are destroyed. While the control variable *u*_3_ is the effort due to eradication through aerial insecticide spraying. Here, we consider that the death rate of the population of adult vector (beetle) increases as *u*_3_ increases. The factor (1 − *u*_3_) measure the reduction in the population of adult beetles. We present the control model based on the assumptions and extensions made above,

17 $$\{\begin{array}{rcl}\frac{d{S}_{H}}{dt} & = & {{\rm{\Lambda }}}_{H}+c{N}_{H}-{\kappa }_{1}\psi {S}_{H}{I}_{V}\mathrm{(1}-{u}_{1})-{\kappa }_{2}\varphi \alpha {S}_{H}{I}_{V}\mathrm{(1}-{u}_{1})-{d}_{1}{S}_{H},\\ \frac{d{E}_{H}}{dt} & = & {\kappa }_{1}\psi {S}_{H}{I}_{V}\mathrm{(1}-{u}_{1})+{\kappa }_{2}\varphi \alpha {S}_{H}{I}_{V}\mathrm{(1}-{u}_{1})-({d}_{1}+\delta ){E}_{H},\\ \frac{d{I}_{H}}{dt} & = & \delta {E}_{H}-({d}_{1}+\gamma ){I}_{H}-{u}_{2}{I}_{H},\\ \frac{d{S}_{V}}{dt} & = & {{\rm{\Lambda }}}_{V}{N}_{V}\mathrm{(1}-{u}_{3})-\mathrm{(1}-{u}_{1})\eta {S}_{V}{I}_{H}-{d}_{2}{S}_{V}-{\alpha }_{o}{u}_{3}{S}_{V},\\ \frac{d{E}_{V}}{dt} & = & \mathrm{(1}-{u}_{1})\eta {S}_{V}{I}_{H}-({d}_{2}+\mu ){E}_{V}-{\alpha }_{o}{u}_{3}{E}_{V},\\ \frac{d{I}_{V}}{dt} & = & \mu {E}_{V}-{d}_{2}{I}_{V}-{\alpha }_{o}{u}_{3}{I}_{V}\mathrm{.}\end{array}$$

subject to non-negative initial conditions.

In system (17), we use three control variables $u(t)=({u}_{1},{u}_{2},{u}_{3})\in {\mathscr{U}}$, which is define briefly above. The control variables $u(t)=({u}_{1},{u}_{2},{u}_{3})\in {\mathscr{U}}$ associate to the variables *S*_*H*_, *E*_*H*_, *I*_*H*_, *S*_*V*_, *E*_*V*_ and *I*_*V*_ are bounded and measure with

18 $$\begin{array}{l}{\mathscr{U}}=\{({u}_{1},{u}_{2},{u}_{3})|{u}_{i}\,is\,Lebsegue\,measurable\,on\,[0,1],\,0\le {u}_{i}(t)\le 1,\,t\in [0,T],\\ \,\,i=1,2,3\}.\end{array}$$

Here, for the control problem we defined the objective functional as

19 $$J({u}_{1},{u}_{2},{u}_{3})={\int }_{0}^{T}\,[{C}_{1}{I}_{h}+{C}_{2}{N}_{v}+\frac{1}{2}({B}_{1}{u}_{1}^{2}+{B}_{2}{u}_{2}^{2}+{B}_{3}{u}_{3}^{2})]dt$$

subject to the control system (17). The constants in (19), *C*_1_, *C*_2_, *B*_1_, *B*_2_ and *B*_3_ are the weight and balancing constants. These constants *C*_1_, *C*_2_ and *B*_1_, *B*_2_, *B*_3_ measure respectively the relative cost of the interventions over the interval [0, *T*]. In order to find an optimal control, ${u}_{1}^{\ast },{u}_{2}^{\ast },{u}_{3}^{\ast }$, such that

20 $$J({u}_{1}^{\ast },{u}_{2}^{\ast },{u}_{3}^{\ast })=\mathop{{\rm{\min }}}\limits_{{\mathscr{U}}}\,J({u}_{1},{u}_{2},{u}_{3})$$

where ${\mathscr{U}}$ is defined in (18) and subject to control system (17) with non-negative initial conditions. Next, we use the well known Pontryagin’s Maximum Principle to find the solution to the control problem and derive its necessary conditions. Here, we show the existence for the control system (control problem). Let the variables *S*_*H*_(*t*), *E*_*H*_(*t*), *I*_*H*_(*t*), *S*_*V*_(*t*), *E*_*V*_(*t*) and *I*_*V*_(*t*) be represent the state variables with control associated control variables *u*_1_(*t*), *u*_2_(*t*) and *u*_3_(*t*). For existence, we rewrite the control problem (17) in the following form:

21 $${\mathscr{X}}^{\prime} =B{\mathscr{X}}+F({\mathscr{X}})$$

where

$${\mathscr{X}}=(\begin{array}{c}{S}_{H}(t)\\ {E}_{H}(t)\\ {I}_{H}(t)\\ {S}_{V}(t)\\ {E}_{V}(t)\\ {I}_{V}(t)\end{array}),$$

and

$$B=(\begin{array}{cccccc}-{d}_{1}+c & c & c & 0 & 0 & 0\\ 0 & -({d}_{1}+\delta ) & 0 & 0 & 0 & 0\\ 0 & \delta  & -({d}_{1}+\gamma +{u}_{2}) & 0 & 0 & 0\\ 0 & 0 & 0 & {{\rm{\Lambda }}}_{V}\mathrm{(1}-{u}_{3})-{d}_{2}-{\alpha }_{o}{u}_{3} & {{\rm{\Lambda }}}_{V}\mathrm{(1}-{u}_{3}) & {{\rm{\Lambda }}}_{V}\mathrm{(1}-{u}_{3})\\ 0 & 0 & 0 & 0 & -({d}_{2}+\mu +{\alpha }_{o}{u}_{3}) & 0\\ 0 & 0 & 0 & 0 & \mu  & -({d}_{2}+{\alpha }_{o}{u}_{3})\end{array}),$$

$$F({\mathscr{X}})=(\begin{array}{c}{{\rm{\Lambda }}}_{H}-{\kappa }_{1}\psi {S}_{H}{I}_{V}\mathrm{(1}-{u}_{1})-{\kappa }_{2}\varphi \alpha {S}_{H}{I}_{V}\mathrm{(1}-{u}_{1})\\ {\kappa }_{1}\psi {S}_{H}{I}_{V}\mathrm{(1}-{u}_{1})+{\kappa }_{2}\varphi \alpha {S}_{H}{I}_{V}\mathrm{(1}-{u}_{1})\\ 0\\ -\eta {S}_{V}{I}_{H}\\ \eta {S}_{V}{I}_{H}\\ 0\end{array})$$

and ${\mathscr{X}}^{\prime}$ represents the derivative with respect to time *t*. The system (21) is a nonlinear system with bounded coefficient. By setting

22 $${\mathscr{G}}({\mathscr{X}})=B{\mathscr{X}}+F({\mathscr{X}})$$

The term *F* $({\mathscr{X}})$ on right hand side of (22) satisfies

23 $$\begin{array}{rcl}|F({{\mathscr{X}}}_{1})-F({{\mathscr{X}}}_{2})| & \le  & {Z}_{1}(|{S}_{1H}(t)-{S}_{2H}(t)|+{Z}_{2}|{E}_{1H}(t)-{E}_{2H}(t)|\\  &  & +\,{Z}_{3}|{E}_{1H}(t)-{E}_{2H}(t)|+{Z}_{4}|{S}_{1V}(t)-{S}_{2V}(t)|\\  &  & +\,{Z}_{5}|{E}_{1V}(t)-{E}_{2V}(t)|+{Z}_{6}|{I}_{1V}(t)-{I}_{2V}(t)|)\\  & \le  & Z(|{S}_{1H}(t)-{S}_{2H}(t)|+|{E}_{1H}(t)-{E}_{2H}(t)|\\  &  & +\,|{E}_{1H}(t)-{E}_{2H}(t)|+|{S}_{1V}(t)-{S}_{2V}(t)|\\  &  & +\,{Z}_{5}|{E}_{1V}(t)-{E}_{2V}(t)|+|{I}_{1V}(t)-{I}_{2V}(t)|)\end{array}$$

where *Z* is the positive constant, *Z* = *max*(*Z*_1_, *Z*_2_, *Z*_3_, *Z*_4_, *Z*_5_, *Z*_6_) is independent of the state variables. Also we have

24 $$|G({{\mathscr{X}}}_{1})-G({{\mathscr{X}}}_{2})|\le Z|{{\mathscr{X}}}_{1}-{{\mathscr{X}}}_{2}|,$$

where *Z* = *Z*_1_ + *Z*_2_ + *Z*_3_ + *Z*_4_ + *Z*_5_ + *Z*_6_ + ||*K*|| < ∞. Thus, it follows that the function *G* $({\mathscr{X}})$ is uniformly Lipschitz continuous. From the definition of control variables we can see that a solution of the system (17) exists^41^.

### Existence of the control problem

Following the result in ref. 42 we prove the existence of an optimal control problem. In control system (17), the equations are obviously bounded above and so the result in ref. 42 can be applied to the model (17), since the state variables and the set of control variables are nonempty, the set ${\mathscr{U}}$ of the control variables is closed and convex. The right hand side of each equation in control problem (17) is continuous, bounded above by a sum of the bounded control and state, and can be written as a linear function of *u* with coefficients depending on time and state and there exists constants *l*_1_, *l*_2_ > 0 and *m* > 1 such that the integrand *L*(*y*, *u*, *t*) of the objective functional *J* is convex and satisfies

$$L(y,u,t)\ge {l}_{1}{({|{u}_{1}|}^{2}+{|{u}_{2}|}^{2}+{|{u}_{3}|}^{2})}^{\frac{m}{2}}-{l}_{2}.$$

To satisfy the conditions mentioned above, we use the result given in ref. 43 to establish the existence of (17). The state variables and the set of control is obviously bounded and nonempty. The solutions are bounded, and convex. The system is bilinear in control variables (since the solutions are bounded). The last one can be verified as

$${C}_{1}{I}_{H}+{C}_{2}{N}_{v}+\frac{1}{2}({B}_{1}{u}_{1}^{2}+{B}_{2}{u}_{2}^{2}+{B}_{3}{u}_{3}^{2})\ge {l}_{1}{({|{u}_{1}|}^{2}+{|{u}_{2}|}^{2}+{|{u}_{3}|}^{2})}^{\frac{m}{2}}-{l}_{2}.$$

where *C*_1_, *C*_2_, *B*_1_, *B*_2_, *B*_3_, *l*_1_, *l*_2_ > 0 and *m* > 1. So, we get

**Theorem 0.6**. *The objective functional* (*19*) *and the control set* (*18*) *subject to control system* (*17*) *there exists an optimal control* ${u}^{\ast }=({u}_{1}^{\ast },{u}_{2}^{\ast },{u}_{3}^{\ast })$ *such that* $J({u}_{1}^{\ast },{u}_{2}^{\ast },{u}_{3}^{\ast })={{\rm{\min }}}_{{\mathscr{U}}}\,J({u}_{1},{u}_{2},{u}_{3})$.

In order to obtain the solution to the control problem, it is necessary to get the Lagrangian and Hamiltonian (17). Hence, the Lagrangian *L* for the control problem is expressed as

25 $$L({I}_{H},{N}_{V},{u}_{1},{u}_{2},{u}_{3})={C}_{1}{I}_{H}+{C}_{2}{N}_{V}+\frac{1}{2}({B}_{1}{u}_{1}^{2}+{B}_{2}{u}_{2}^{2}+{B}_{1}{u}_{3}^{2})$$

and for the Hamiltonian *H*, by choosing $\underline{X}=({S}_{H},{E}_{H},{I}_{H},{S}_{V},{E}_{V},{I}_{V})$, ${\mathscr{U}}=({u}_{1},{u}_{2},{u}_{3})$ and *λ* = (*λ*_1_, *λ*_2_, *λ*_3_, *λ*_4_, *λ*_5_, *λ*_6_), we have:

26 $$\begin{array}{rcl}H(\underline{X},{\mathscr{U}},\lambda ) & = & L({I}_{H},{N}_{V},{u}_{1},{u}_{2},{u}_{3})+{\lambda }_{1}[{\Lambda }_{H}+c{N}_{H}-{\kappa }_{1}\psi {S}_{H}{I}_{V}\mathrm{(1}-{u}_{1})\\  &  & -{\kappa }_{2}\varphi \alpha {S}_{H}{I}_{V}\mathrm{(1}-{u}_{1})-{d}_{1}{S}_{H}]+{\lambda }_{2}[{\kappa }_{1}\psi {S}_{H}{I}_{V}\mathrm{(1}-{u}_{1})\\  &  & +\,{\kappa }_{2}\varphi \alpha {S}_{H}{I}_{V}\mathrm{(1}-{u}_{1})-({d}_{1}+\delta ){E}_{H}]+{\lambda }_{3}[\delta {E}_{H}-({d}_{1}+\gamma ){I}_{H}\\  &  & -{u}_{2}{I}_{H}]+{\lambda }_{4}[{\Lambda }_{V}{N}_{V}\mathrm{(1}-{u}_{3})-\eta {S}_{V}{I}_{H}\mathrm{(1}-{u}_{1})\\  &  & -{d}_{2}{S}_{V}-{\alpha }_{o}{u}_{3}{S}_{V}]+{\lambda }_{5}[\eta {S}_{V}{I}_{H}\mathrm{(1}-{u}_{1})-({d}_{2}+\mu ){E}_{V}\\  &  & -{\alpha }_{o}{u}_{3}{E}_{V}]+{\lambda }_{6}[\mu {E}_{V}-{d}_{2}{I}_{V}-{\alpha }_{o}{u}_{3}{I}_{V}\mathrm{].}\end{array}$$

### Solution to the Optimal control problem

To obtain the optimal solution of the control system (17), we use the well-known Pontryagin’s Maximum Principle^44^:

Letting ${u}_{1}^{\ast }$, ${u}_{2}^{\ast }$ and ${u}_{3}^{\ast }$ represent the solutions to the control problem (17), then, there exists the adjoint variables *λ*_*i*_ for *i* = 1, 2, 3, 4, 5, 6 satisfying the following conditions given below.

27 $$\begin{array}{rcl}\,\frac{dx}{dt} & = & \frac{\partial H(t,{u}_{1}^{\ast },{u}_{2}^{\ast },{u}_{3}^{\ast },{\lambda }_{1},{\lambda }_{2},{\lambda }_{3},{\lambda }_{4},{\lambda }_{5},{\lambda }_{6})}{\partial \lambda },\\ \,\,\,\,\,0 & = & \frac{\partial H(t,{u}_{1}^{\ast },{u}_{2}^{\ast },{u}_{3}^{\ast },{\lambda }_{1},{\lambda }_{2},{\lambda }_{3},{\lambda }_{4},{\lambda }_{5},{\lambda }_{6})}{\partial u},\\ \frac{d\lambda }{dt} & = & -\frac{\partial H(t,{u}_{1}^{\ast },{u}_{2}^{\ast },{u}_{3}^{\ast },{\lambda }_{1},{\lambda }_{2},{\lambda }_{3},{\lambda }_{4},{\lambda }_{5},{\lambda }_{6})}{\partial x}\mathrm{.}\end{array}$$

Here, by applying the necessary conditions to the Hamiltonian H, the following is obtained:

**Theorem 0.7**. *The given optimal controls* ${u}_{1}^{\ast },{u}_{2}^{\ast },{u}_{3}^{\ast }$ *and the solutions* ${S}_{H}^{\ast },{E}_{H}^{\ast },{I}_{H}^{\ast },{S}_{V}^{\ast },{E}_{V}^{\ast },{I}_{V}^{\ast }$ *of the state system*, *there exists the adjoint variables λ*_*i*_ *for i* = 1, 2, 3, 4, 5, 6

28 $$\begin{array}{rcl}{\lambda }_{1}^{^{\prime} } & = & -{\lambda }_{1}c+{\lambda }_{1}{d}_{1}+({\lambda }_{1}-{\lambda }_{2}){\kappa }_{1}\psi {I}_{V}\mathrm{(1}-{u}_{1})+({\lambda }_{1}-{\lambda }_{2}){\kappa }_{2}\varphi \alpha {I}_{V}\mathrm{(1}-{u}_{1}),\\ {\lambda }_{2}^{^{\prime} } & = & -{\lambda }_{1}c-{\lambda }_{3}\delta +{\lambda }_{2}({d}_{1}+\delta ),\\ {\lambda }_{3}^{^{\prime} } & = & -{C}_{1}+\eta {S}_{V}\mathrm{(1}-{u}_{1})({\lambda }_{4}-{\lambda }_{5})+{\lambda }_{3}{u}_{2}+{\lambda }_{3}({d}_{1}+\gamma )-{\lambda }_{1}c,\\ {\lambda }_{4}^{^{\prime} } & = & -{C}_{2}-{{\rm{\Lambda }}}_{V}{\lambda }_{4}\mathrm{(1}-{u}_{3})+({\lambda }_{4}-{\lambda }_{5})\eta {I}_{H}\mathrm{(1}-{u}_{1})+{d}_{2}{\lambda }_{4}+{\lambda }_{4}{\alpha }_{o}{u}_{3},\\ {\lambda }_{5}^{^{\prime} } & = & -{C}_{2}-{{\rm{\Lambda }}}_{V}{\lambda }_{4}\mathrm{(1}-{u}_{3})+{\lambda }_{5}({d}_{2}+\mu )+{\lambda }_{5}{\alpha }_{o}{u}_{3}-{\lambda }_{6}\mu ,\\ {\lambda }_{6}^{^{\prime} } & = & -{C}_{2}+({\lambda }_{1}-{\lambda }_{2}){\kappa }_{1}\psi {S}_{H}\mathrm{(1}-{u}_{1})+({\lambda }_{1}-{\lambda }_{2}){\kappa }_{2}\varphi \alpha {S}_{H}\mathrm{(1}-{u}_{1})\\  &  & -{\lambda }_{4}{{\rm{\Lambda }}}_{V}\mathrm{(1}-{u}_{3})+{\lambda }_{6}({d}_{2}+{\alpha }_{o}{u}_{3}),\end{array}$$

*with transversality conditions*

29 $${\lambda }_{1}({T}_{f})={\lambda }_{2}({T}_{f})={\lambda }_{3}({T}_{f})={\lambda }_{4}({T}_{f})={\lambda }_{5}({T}_{f})={\lambda }_{6}({T}_{f})=0.$$

*Further*, *the controls*, ${u}_{1}^{\ast },{u}_{2}^{\ast },{u}_{3}^{\ast }$ *are given by*

30 $$\begin{array}{rcl}{u}_{1}^{\ast } & = & {\rm{\max }}\{{\rm{\min }}\{\mathrm{1,}\tfrac{({\lambda }_{2}-{\lambda }_{1})({\kappa }_{1}\psi {S}_{H}^{\ast }{I}_{V}^{\ast }+{\kappa }_{2}\varphi \alpha {S}_{H}^{\ast }{I}_{V}^{\ast })+({\lambda }_{5}-{\lambda }_{4})\eta {S}_{V}^{\ast }{I}_{H}^{\ast }}{{B}_{1}}\},0\},\\ {u}_{2}^{\ast } & = & {\rm{\max }}\{{\rm{\min }}\{\mathrm{1,}\tfrac{{\lambda }_{3}{I}_{H}^{\ast }}{{B}_{2}}\},0\},\\ {u}_{3}^{\ast } & = & {\rm{\max }}\{{\rm{\min }}\{\tfrac{{\lambda }_{4}({{\rm{\Lambda }}}_{V}{N}_{V}+{\alpha }_{o}{S}_{V}^{\ast })+{\alpha }_{o}({\lambda }_{5}{E}_{V}^{\ast }+{\lambda }_{6}{I}_{V}^{\ast })}{{B}_{3}}\}\mathrm{,0}\}\end{array}$$

**Proof:** To find the adjoint system (28) and the transversality conditions (29), we use *H* (26) by setting ${S}_{H}={S}_{H}^{\ast }$, ${E}_{H}={E}_{H}^{\ast }$, ${I}_{H}={I}_{H}^{\ast }$, ${S}_{V}={S}_{V}^{\ast }$, ${E}_{V}={E}_{V}^{\ast }$ and ${I}_{V}={I}_{V}^{\ast }$ and taking the time derivative of *H* with respect to *S*_*H*_, *E*_*H*_, *I*_*H*_, *S*_*V*_, *E*_*V*_, *I*_*V*_, we obtain (28). To find the optimal control characterization (30), we use $\frac{\partial H}{\partial {u}_{i}}=0$, for *i* = 1, 2, 3.

## Numerical Simulations and Results

Here, we are investigating the numerical solutions of the system (17) and that of the model without control (1). For the solution of both systems (that is the control and without control) many method are available in the literature^45, 46^. In this simulation, the cases without control population are labeled with bold line and the control cases by a dashed line. The constants (weight) values in the objective functional are *C*_1_ = 0.01, *C*_2_ = 0.0036, *B*_1_ = 0.02, *B*_2_ = 0.3, *B*_3_ = 3. The parameters values used in the optimal control solution is chosen in such a way, that the number of infected trees, exposed trees, susceptible vector, exposed vector and infected vector decreased while the population of susceptible trees increased. These are given in Table 1. We adopted different control strategies to minimize the infection in pine trees population by considering $({u}_{1}=0,{u}_{2}\ne 0,{u}_{3}\ne 0)$, $({u}_{1}\ne \mathrm{0,}{u}_{2}=\mathrm{0,}{u}_{3}\ne \mathrm{0)}$, $({u}_{1}\ne \mathrm{0,}{u}_{2}\ne 0,{u}_{3}=\mathrm{0)}$, and $({u}_{1}\ne \mathrm{0,}{u}_{2}\ne \mathrm{0,}{u}_{3}\ne \mathrm{0)}$.

**Table 1:** Values of parameter used in numerical simulation of the optimal control system.

Notation	Value	References
Λ_*H*_	0.002021/day	Assumed
c	0.001241/day	Assumed
*κ*_1_	0.00166/day	48
*κ*_2_	0.0004/day	48
*ψ*	0.20/day	49
*γ*	0.00220/day	Assumed
*d*_1_	0.0000301/day	50
*δ*	0.0133/day	Assumed
*α*	0.0032/day	assumed
*η*	0.00305/day	51
*μ*	0.01/day	Assumed
Λ_*V*_	0.0132652/day	Assumed
*α*_*o*_	0.21/day	Assumed
*ϕ*	0.0023/day	Assumed
*d*_2_	0.011764/day	52

**Figure 2:**
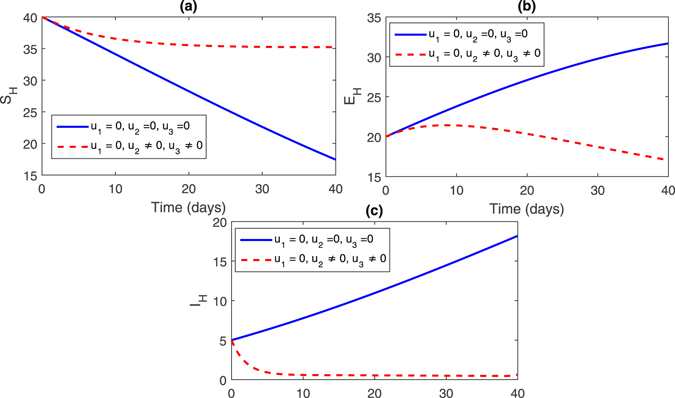
Comparison of both the systems: without and with control.

**Figure 3:**
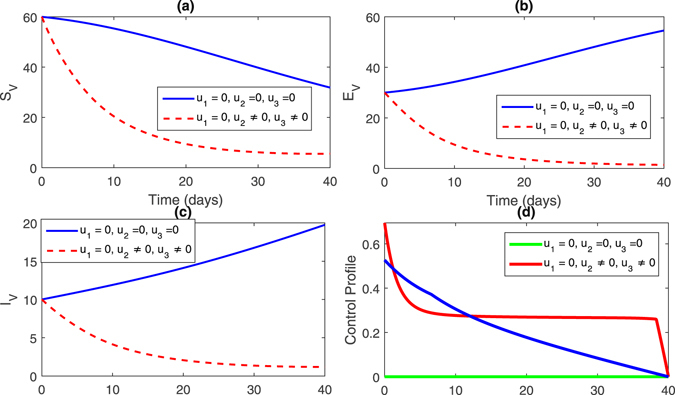
Comparison of both the systems: without and with control.

**Strategy 1:** $({u}_{1}=\mathrm{0,}{u}_{2}\ne \mathrm{0,}{u}_{3}\ne \mathrm{0)}$ In this strategy, we set the control variable *u*_1_ = 0 zero and the rest of the control variables are non-zero, this effect can bee seen in Fig. 2, (sub-figures (a–c)) and Fig. 3 (sub-figures, (a–d)). The population of susceptible trees increased while the exposed, infected trees and vector population decreased.

**Figure 4:**
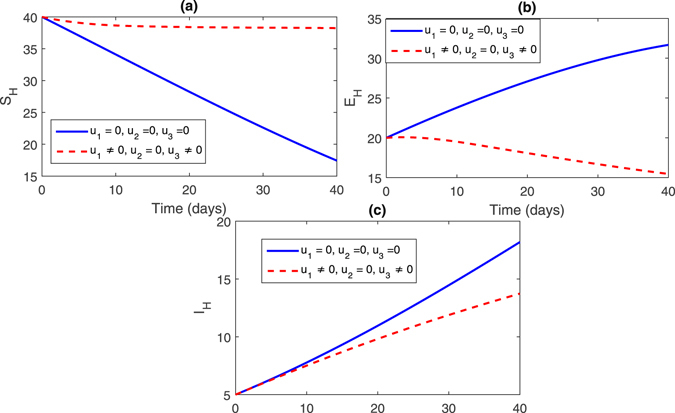
Comparison of both the systems: without and with control.

**Figure 5:**
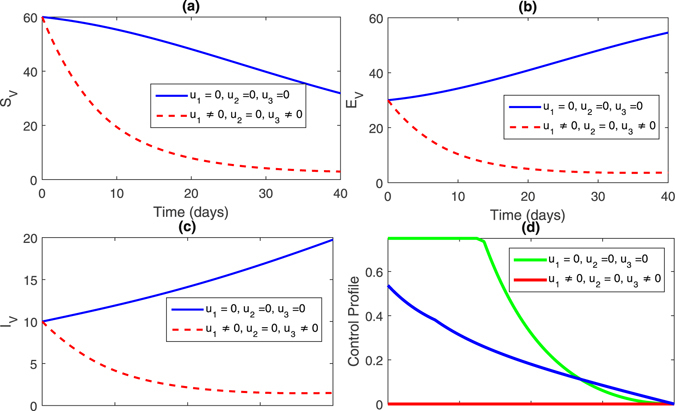
Comparison of both the systems: without and with control.

**Strategy 2:** $({u}_{1}\ne \mathrm{0,}{u}_{2}=\mathrm{0,}{u}_{3}\ne \mathrm{0)}$ In this strategy, we set the control variable *u*_2_ = 0 and the rest of the control variables are non-zero, this impact can bee seen in Fig. 4, (sub-figures (a–c)) and Fig. 5 (sub-figures, (a–d)). The population of susceptible trees increased while the exposed, infected trees and vector population decreased. One can observe in Figs 2(c) and 4(c), that the infected trees population did not decrease compare to strategy 1.

**Figure 6:**
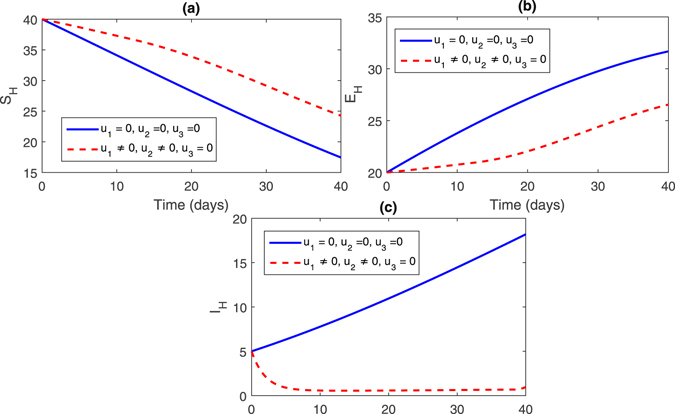
Comparison of both the systems: without and with control.

**Figure 7:**
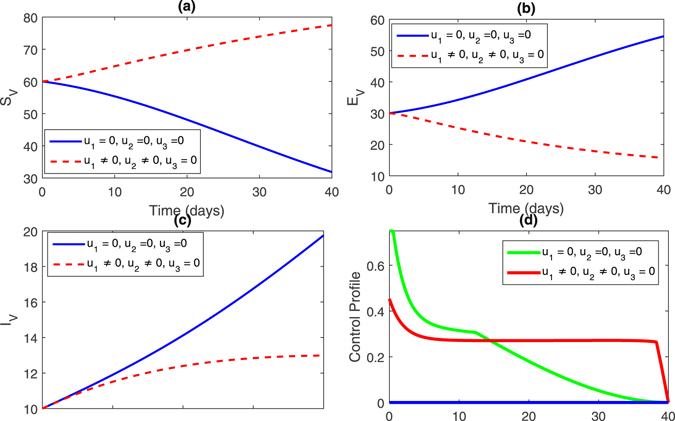
Comparison of both the systems: without and with control.

**Strategy 3:** $({u}_{1}\ne \mathrm{0,}{u}_{2}\ne \mathrm{0,}{u}_{3}=\mathrm{0)}$ In this strategy, we set the control variable *u*_3_ = 0 and the rest of the control variables are non-zero, this effect can bee seen in Fig. 6, (sub-figures (a–c)) and Fig. 7 (sub-figures, (a–d)). The population of susceptible trees increased sharply while the exposed, infected trees and vector population decreased. One can see that infected population significantly reduced, see Fig. 6(c).

**Figure 8:**
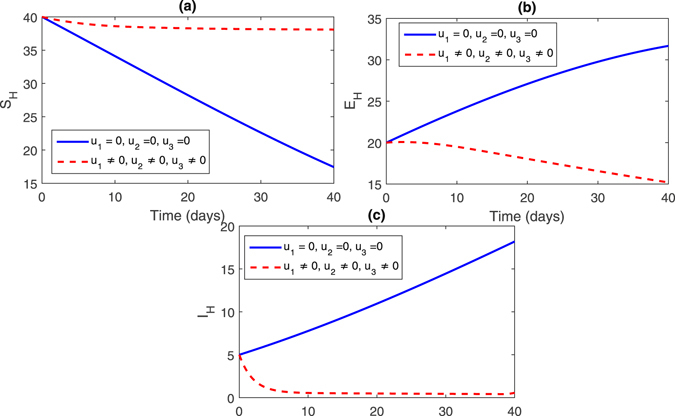
Comparison of both the systems: without and with control.

**Figure 9:**
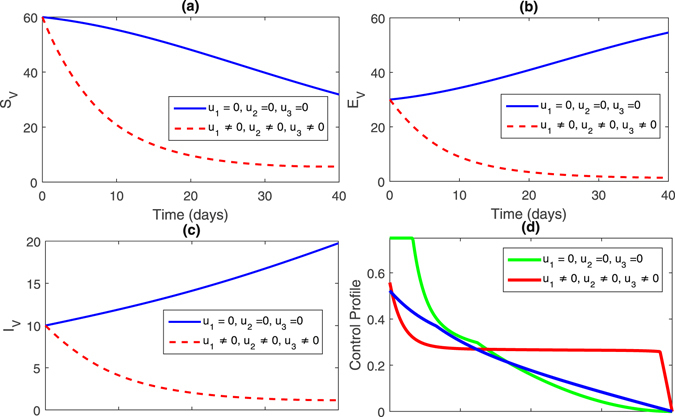
Comparison of both the systems: without and with control.

**Strategy 4:** $({u}_{1}\ne 0,{u}_{2}\ne 0,{u}_{3}\ne \mathrm{0)}$ In this strategy, we activate all the control. The figures for this strategy can be seen in Fig. 8, (sub-figures (a–c)) and Fig. 9 (sub-figures, (a–d)). The population of susceptible trees increased, while the exposed, infected trees and vector population decreased. Thus, by comparing all the control stratigies from 1–4, one can observe that the strategy 4 is the best strategy to control infections in the pine trees population.

## Conclusion

We have considered a mathematical system of equation which describes the pine wilt disease. The analysis of the system is well established. The stability analysis of the disease free and endemic equilibria is presented on the basis of basic reproduction number ${ {\mathcal R} }_{0}$. Whenever the basic reproduction number ${ {\mathcal R} }_{0} < 1$, the disease free equilibrium is stable both locally and globally. If the basic reproduction number ${ {\mathcal R} }_{0} > 1$, then the endemic equilibrium equilibrium is stable both locally and globally. A bifurcation analysis of the model is presented and a control problem is formulated by using three control variables, two control for pine trees population and one control for vector population. The mathematical results for the control problem with different control strategies are presented and concluded that the strategy 4 is the best control strategy for cost minimization in the population of pine trees diseases. This work generalized the work presented in ref. 21 by incorporating the exploded class *E*_*V*_ in the vector population. In ref. 21, the optimal control solution is investigated without defining the control strategies. In this new work, we suggests different control strategies for the eradication of infection in Pine trees. So, this work compared to the previous study is more helpful for the elimination of pine trees infections^47^.
